# Prevalence of prediabetes and risk of CVD mortality in individuals with prediabetes alone or plus hypertension in Northeast China: insight from a population based cohort study

**DOI:** 10.1186/s12889-024-17996-y

**Published:** 2024-02-15

**Authors:** Ling Yue, Yuanmeng Tian, Mingxi Ma, Li Jing, Qun Sun, Lei Shi, Jixu Sun, Weizhong Wang, Guangxiao Li, Liying Xing, Shuang Liu

**Affiliations:** 1 Department of Ultrasound, The Fourth Hospital of China Medical University, Shenyang, Liaoning China; 2 Institute of Preventive Medicine, China Medical University, Shenyang, Liaoning China; 3 Department of Chronic Disease Preventive and Control, Liaoning Provincial Center for Disease Control and Prevention, Shenyang, Liaoning China; 4 Department of Chronic Disease, Disease Control and Prevention of Chao Yang City, Chaoyang, Liaoning China; 5 Department of Chronic Disease, Disease Control and Prevention of Liao Yang City, Liaoyang, Liaoning China; 6 Department of Chronic Disease, Disease Control and Prevention of Dan Dong City, Dandong, Liaoning China; 7 Department of Neurology, Central Hospital of Dan Dong City, Dandong, Liaoning China; 8 Department of Medical Record Management Center, The First Affiliated Hospital of China Medical University, Shenyang, Liaoning China

**Keywords:** China, Cardiovascular mortality, Prediabetes, Hypertension

## Abstract

**Background:**

To evaluate the current prevalence of prediabetes in northeast China, and further determine the association between prediabetes alone or coexistent with hypertension and cardiovascular disease (CVD) mortality.

**Methods:**

In the prospective study, 15,557 participants without diabetes among aged ≥40 years in northeast China, were followed for a median of 5.5 years. Following the American Diabetes Association, prediabetes was defined as fasting plasma glucose (FPG) range of 5.6-6.9 mmol/L or glycated hemoglobin (HbA1c) range of 5.7-6.4% in people without diabetes.

**Results:**

The prevalence of prediabetes was 44.3% among population aged ≥40 years in northeast China. Prediabetes alone did not promote risk of CVD mortality. However, when the subgroups were stratified by hypertension, the CVD mortality risk in prediabetes plus hypertension subjects increased significantly compared with population without prediabetes and hypertension. Multivariate-adjusted hazard ratios for CVD mortality in prediabetes subgroups plus hypertension were 2.28 (95% CI: 1.50, 3.47) for those diagnosed by FPG < 5.6 mmol/L & HbA1c 5.7-6.4%, 2.18 (95% CI: 1.53, 3.10) for those diagnosed by FPG 5.6-6.0 mmol/L & HbA1c < 6.5% and 2.35 (95% CI: 1.65, 3.35) for those diagnosed by FPG 6.1-6.9 & HbA1c < 6.5% compared with the reference group. Moreover, the percentage of hypertension in prediabetes subjects was high (60.4%), but the awareness, treatment and control rates were far from satisfactory (45.3, 35.1 and 4.8%, respectively).

**Conclusions:**

The prevalence of prediabetes remains high in northeast China, and the CVD mortality was elevated significantly in prediabetes coexistent with hypertension. Considering the high percentage and low control rate of hypertension in prediabetes, strategies focused on HbA1c screening, FPG lowering and blood pressure management should be emphasized in northeast China.

**Supplementary Information:**

The online version contains supplementary material available at 10.1186/s12889-024-17996-y.

## Background

Prediabetes refers to blood glucose concentrations higher than normal but lower than diabetic thresholds, is a high-risk state for progressing to diabetes. The prevalence of prediabetes is rising globally, a previous study has projected that more than 470 million individuals will have prediabetes by 2030 [1]. In China, possibly due to rapid socio-demographic changes and health transitions, the prevalence of prediabetes has reached 35.7% in 2016, [2] which was relatively higher than that in many other countries, [3] despite the conversion rate differs with prediabetes criteria and population characteristics [4].

Approximately 5-10% of people with prediabetes develop diabetes each year, which is much higher than the 3.5% in the general population, [5] In addition, prediabetes itself is also a risk factor for cardiovascular and cerebrovascular disease (CVD). Previous studies have suggested that prediabetes alters vascular status, worsens the prognosis of stroke, [6, 7] significantly increases the morbidity and mortality [8].

Moreover, the Jackson Heart Study indicated the associations between prediabetes and risk of CVD and mortality may be explained by concomitant hypertension [9]. Participants with both prediabetes and hypertension experienced a higher risk than those with prediabetes or hypertension alone [9]. According to the World Health Organization (WHO), prediabetes is characterized by fasting plasma glucose (FPG) of 6.1-6.9 mmol/L [10]. However, the American Diabetes Association (ADA) uses a lower cutoff value (FPG, 5.6-6.9 mmol/L) and has additionally introduced hemoglobin A1c (HbA1c) levels of 5.7-6.4% to diagnose prediabetes [11]. Compared with normoglycemic populations, the risk of CVD and mortality was related to hemoglobin A1c (HbA1c) 5.7-6.4% defined prediabetes (relative risk, 1.17), but not FPG (5.6-6.0 mmol/L, 6.1-6.9 mmol/L or 5.6-6.9 mmol/L) according to the Whitehall II study [12].

However, previous epidemiological studies on prediabetes have been criticized because of single diagnostic index, [13, 14] or lack of adverse health outcomes [9]. Therefore, we conducted a prospective study to profile the characteristics of prediabetes according to ADA recommendations, and further analyze the CVD mortality in different phenotypes of prediabetes as well as coexistent with hypertension, aiming to provide population-based evidence for formulating strategies to alleviate CVD burden.

## Methods

### Subjects and study design

This prospective cohort study is based on a cross-sectional study which was conducted between September 2017 and March 2019 in northeast China. The design of the study has been described previously. In brief, a total of 18,796 participants aged 40 years and older were enrolled from four rural counties (Donggang, Lingyuan, Chaoyang and Liaoyang) and three urban districts (Zhennan, Liuerbao and Gongchangling) in Liaoning Province, using a multi-stage stratified cluster random sampling method. Detailed information was collected at baseline for each participant, the detailed information has been described previously [15].

Subsequently, individuals with diabetes or missing information on FPG or HbA1c were excluded, a total of 15,557 subjects were enrolled finally. The study was approved by the Ethics Commission of the CPC Central Committee of the China Cardiovascular Disease Center (Beijing). All participants provided written informed consent. Patients and public will not be involved in the development of the research question or in the design of the study. Patients will receive written information about this trial.

### Data collection

Data is in a single clinic visit, through face-to-face interview using self-management questionnaire to collect. Take fasting blood samples in the morning after overnight fasting ≥8 hours. Samples were injected from the anterior elbow vein into BD Vacutainer tubes containing EDTA (Becton, Dickinson and Co, Franklin Lakes, New Jersey, USA). Serum samples from whole blood separation, at 20 °C-cryopreservation. FPG was measured by using oxidase enzymatic method on an Abbott Diagnostics C800i auto-analyzer (Abbott Laboratories, Abbott Park, IL, USA) with commercial kits. HbA1c was measured by using quantitative high-performance liquid chromatography from venous blood samples directly.

Blood pressure was measured by a standardized electronic sphygmomanometer (HEM-907; Omron, Kyoto, Japan) in a quiet and warm room. The participants were asked to avoid smoking, caffeine intake and exercise for at least 30 minutes before the measurement. For each participant, blood pressure was measured three times at 2-min intervals after at least 5 min of rest in a seat position. Information on demographic data, medication and lifestyle were obtained from questionnaire.

### Definitions

According to the American Diabetes Association (ADA) guideline, [11] prediabetes was defined as fasting plasma glucose (FPG) range of 5.6-6.9 mmol/L, or HbA1c range of 5.7-6.4% in people without diabetes. Prediabetes was divided into 3 subgroups according to FPG and HbA1c levels: FPG < 5.6 mmol/L & HbA1c 5.7-6.4%, FPG 5.6-6.0 mmol/L & HbA1c < 6.5%, and FPG 6.1-6.9 mmol/L & HbA1c < 6.5%.

Diabetes was identified if the participant met either of the following criteria: (1) self-reported diagnosis that was made by a certified physician previously, (2) an FBG ≥ 7.0 mmol/L or HbA1c ≥ 6.5% according to the ADA guideline. Hypertension was diagnosed if the individual met either of the following criteria: (1) mean systolic blood pressure (SBP) ≥ 140 mmHg and/or mean diastolic blood pressure (DBP) ≥ 90 mmHg, (2) use of antihypertensive medication in the past 2 weeks. Atrial fibrillation (AF) was determined based on electrocardiogram (ECG) findings and/or previous diagnosis by a physician. Current smoking was considered as the consumption of ≥1 cigarette per day and lasted for ≥1 year, while current drinking was defined as any alcohol consumption ≥1 time per week. Regular exercise was defined as moderate-intensity exercise or equivalent to walking for at least 30 minutes and 3 times per week; participants with moderate and heavy manual work were considered to fulfill the criteria. Lack of exercise was identified when participants failed to meet the criteria for regular exercise.

Each participant had a medical history of hypertension. We divided hypertension into 4 subgroups according to severity of hypertension defined by the European Society of Hypertension (ESH) guideline: HBP (normal), HBP(1+), HBP(2+), HBP(3+) [16]. Awareness of hypertension was defined as having an answer of “Yes” to the question “Have you been diagnosed with hypertension by a certified doctor?” Treatment of hypertension was defined as use of anti-hypertensive medication in the past 2 weeks. Hypertension control was defined as an average SBP < 140 mmHg and an average DBP < 90 mmHg, while uncontrolled hypertension was considered as not meeting these criteria.

### Outcomes

Our study endpoint was CVD death. Mortality data was obtained from the National Population Registry of the China National Statistical Office. We accessed the database containing death certificates for CVD deaths that occurred between the cross-sectional study conducted date and September 30, 2023. The cause of death was determined by reviewing the death certificates and classified according to the International Classification of Diseases (10th Revision) codes I00 to I99.

### Statistical analysis

Descriptive statistics were calculated for all variables. Continuous variables with normal distribution were described as means and standard deviations, categorical variables were reported as medians and inter-quartile ranges. Differences between groups were compared with t-test or chi-square test. The direct age- and sex- standardization method was used to evaluate the standardized prevalence according to the 2010 Chinese census, and corresponding 95% confidence intervals (CIs) were estimated.

Cox proportional hazards models were used to estimate associations between subgroups of prediabetes or coexistent with hypertension and the risk of CVD death, hazard ratios (HRs) and 95% CIs were calculated. Model 1 was unadjusted. Model 2 was adjusted for age and sex. Model 3 was further adjusted for body mass index (BMI), history of atrial fibrillation, stroke and heart disease, level of triglyceride (TG), low-density lipoprotein-cholesterol (LDL-C), high-density lipoprotein-cholesterol (HDL-C) and total cholesterol (TC), treatment for hypertension, current smoking, current drinking, education, income, physical activity. All statistical analyses were performed using SPSS 22.0 (SPSS, Chicago, Illinois, USA). *P* value < 0.05 was considered statistically significant.

## Results

### Characteristics of the study population without diabetes

The study included 15,557 subjects without diabetes, with an average age of 59.9 ± 10.1 years, 6115 (39.3%) were males, 72.2% came from rural areas and 32.9% lower income families (< 5000 yuan), half of whom (48.9%) had received education to primary school or below (Table 1).

**Table 1:** Baseline characteristics by normal glycemia and subgroups of prediabetes

Characteristics	Normal glycemia	Prediabetes			
		FPG < 5.6 mmol/L & HbA1c 5.7-6.4%	FPG 5.6-6.0 mmol/L & HbA1c < 6.5%	FPG 6.1-6.9 mmol/L & HbA1c < 6.5%	Total
Participant, n (%)	7249(46.6)	1466(9.4)	3895(25.0)	2947(18.9)	8308(53.4)
Region (%)					
Rural	64.2	66.5	79.3	85.6	79.3
Urban	35.8	33.5	20.7	14.4	20.7
Sex (%)					
Male	37.5	30.2	41.2	45.9	40.9
Female	62.5	69.8	58.8	54.1	59.1
Mean age (y)	58.9 ± 10.3	61.8 ± 9.5	60.3 ± 9.8	61.0 ± 9.9	60.8 ± 9.8
40-49	20.8	9.2	15.4	13.5	13.6
50-59	31.7	31.5	30.8	30.5	30.8
60-69	32.1	39.1	35.9	36	36.5
70-79	12.5	16.5	15.2	17.2	16.1
> = 80	2.9	3.7	2.7	2.8	2.9
Education (%)					
Primary school or lower	45.1	49.4	51	55	52.1
Middle school	40.7	38.7	38.3	35	37.2
High school or above	14.2	11.9	10.7	10	10.7
Annual household income (%)					
< 5000 (yuan)	27.6	32.4	35.7	42.4	37.5
5000-9999 (yuan)	17.7	16.5	19.1	18.4	18.4
10,000-19,999 (yuan)	17.9	17	17.9	17.8	17.7
> = 20,000 (yuan)	36.8	34.1	18.1	21.4	26.4
Current smoking (%)	25.8	20.2	25.7	27.6	25.4
Alcohol drinking (%)	25.5	18.3	28.9	33.2	28.5
Lack of Exercise (%)	11.9	12.9	12.3	13.7	12.9
Hypertension (%)	46.1	53.1	58.4	66.7	60.4
BMI (kg/m^2^)	24.0 ± 3.5	24.8 ± 3.7	24.7 ± 3.6	25.0 ± 3.6	24.9 ± 3.6
SBP (mmHg)	137.2 ± 21.8	141.0 ± 21.5	144.0 ± 21.9	148.3 ± 21.9	145.0 ± 22.0
DBP (mmHg)	84.1 ± 11.5	83.7 ± 11.1	85.7 ± 11.3	87.5 ± 11.7	86.0 ± 11.5
TG (mmol/L)	1.5 ± 1.2	1.7 ± 1.3	1.6 ± 1.4	1.8 ± 1.6	1.7 ± 1.5
TC (mmol/L)	4.9 ± 1.0	5.3 ± 1.1	5.2 ± 1.1	5.3 ± 1.1	5.2 ± 1.1
LDL-C (mmol/L)	2.3 ± 0.9	2.7 ± 1.1	2.6 ± 0.9	2.7 ± 0.9	2.6 ± 0.9
HDL-C (mmol/L)	1.9 ± 0.7	1.8 ± 0.8	1.8 ± 0.7	1.7 ± 0.6	1.8 ± 0.7

*FPG* Fasting plasma glucose, *HbA*1*c* Hemoglobin A1c, *BMI* Body mass index, *SBP* Systolic blood pressure, *DBP* Diastolic blood pressure, *TG* Triglyceride, *TC* Total cholesterol:, *LDL-C* Low-density lipoprotein cholesterol and *HDL-C* High-density lipoprotein cholesterol

**Fig. 1:**
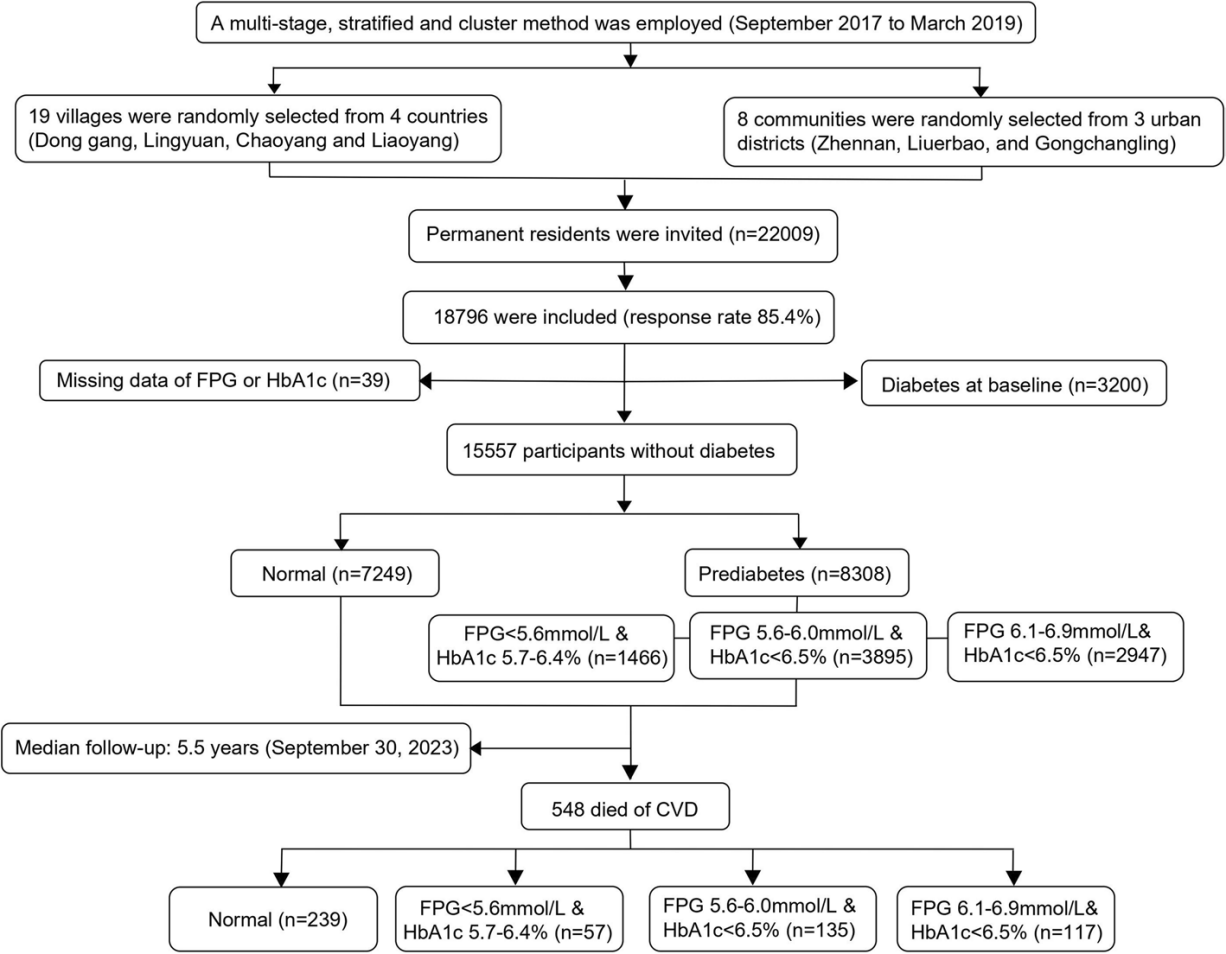
Flow chart of the population selection process

At baseline, 8308 (53.4%) participants were identified to have prediabetes according to ADA definition. Among those subjects, 25.4% were current smokers, 28.5% were current drinkers, 12.9% lacked exercise and 60.4% had hypertension (Table 1) (Fig. 1).

### Prevalence of prediabetes and subgroups

**Fig. 2:**
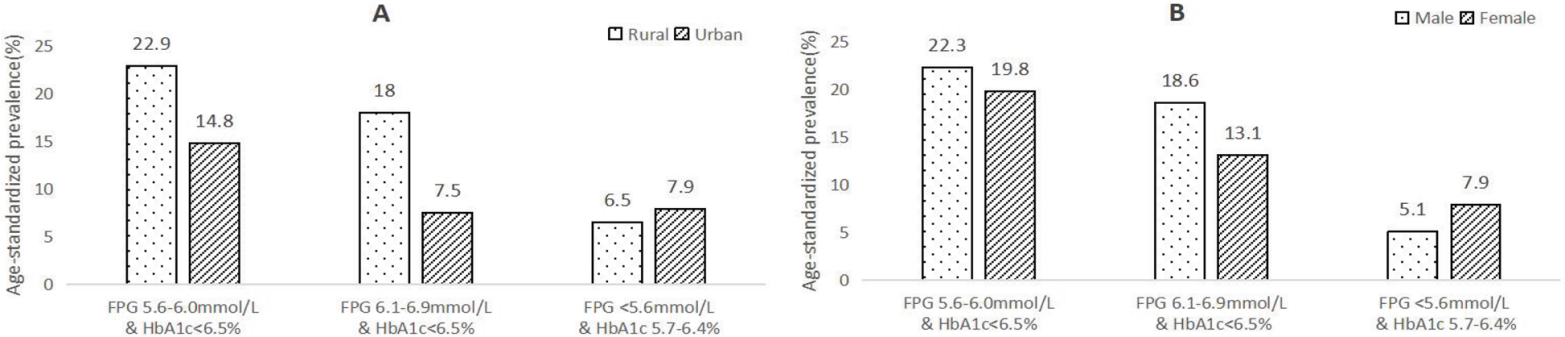
Age-standardized prevalence of prediabetes and subgroups. **A.** Region **B.** Sex

Based on the 2010 ADA criteria, the prevalence of prediabetes was 44.3%, with a higher prevalence in the rural population than in the urban population (49.3% vs 31.8%, *P* < 0.001). We also found that the prevalence in the male group and female group was 46.4 and 42.9%, respectively (*P* < 0.001). The prevalence of prediabetes increased from 39.1% among those 40-49 years to 47.6% among those 70-79 years, however, the prediabetes prevalence decreased slightly among those ≥80 years (45.4%). The age-standardized prevalence was 42.8% (rural 47.4% and urban 30.2%; males 46.0% and females 40.8%) (Table 2) (Fig. 2).

**Table 2:** Prevalence of prediabetes and subgroups in northeast China

Characteristics	Prediabetes [%, (95% CI)]			
	FPG < 5.6 mmol/L & HbA1c 5.7-6.4%	FPG 5.6-6.0 mmol/L & HbA1c < 6.5%	FPG 6.1-6.9 mmol/L & HbA1c < 6.5%	Total
Age group (y)				
40-49	4.7(4.0, 5.5)	20.7(19.2, 22.2)	13.7(12.5, 15.0)	39.1(37.3, 40.9)
50-59	8.0(7.4, 8.8)	20.8(19.8, 21.2)	15.6(14.7, 16.6)	44.5(43.2, 45.8)
60-69	8.5(7.8, 9.2)	20.7(19.8, 21.7)	15.7(14.9, 16.6)	44.9(43.7, 46.1)
70-79	8.6(7.6, 9.7)	21.0(19.5, 22.5)	18.0(16.7, 19.5)	47.6(45.8, 49.5)
> = 80	10.0(7.8, 12.9)	19.9(16.7, 23.5)	15.4(12.6, 18.7)	45.4(41.2, 49.6)
*P*	< 0.001	0.008	< 0.001	< 0.001
Region				
Rural	7.3(6.9, 7.8)	23.1(22.4, 23.9)	18.9 (18.3, 19.6)	49.3(48.5, 50.2)
Urban	9.1(8.3, 9.9)	14.9(14.0, 15.9)	7.8 (7.1, 8.6)	31.8(30.6, 33.0)
*P*	< 0.001	< 0.001	< 0.001	< 0.001
Sex				
Male	6.0(5.5, 6.6)	21.9(21.0, 22.9)	18.5(17.6, 19.4)	46.4(45.3, 47.6)
Female	8.9(8.4, 9.5)	20.0(19.4, 20.8)	14.0(13.3, 14.6)	42.9(42.0, 43.9)
*P*	< 0.001	< 0.001	< 0.001	< 0.001
Total	7.8(7.4, 8.2)	20.8(21.2, 20.4)	15.7(15.2, 16.2)	44.3(43.6, 45.0)
ASR^+^	6.9(6.5, 7.3)	20.8(20.4, 21.2)	15.1(14.6, 15.6)	42.8(39.1, 46.5)

*FPG* fasting plasma glucose: *HbA*1*c* hemoglobin A1c

*ASR* according to the 2010 Chinese census population standardized prevalence rate

In the subgroup analysis, the prevalence of FPG < 5.6 mmol/L & HbA1c 5.7-6.4%, FPG 5.6-6.0 mmol/L & HbA1c < 6.5% and FPG 6.1-6.9 mmol/L & HbA1c < 6.5% was 7.8, 20.8 and 15.7% respectively. Young men and residents living in rural areas were tended to had higher FPG, in contrast, older women and residents living in urban areas were tended to have raised HbA1c (Table 2) (Fig. 2).

### CVD mortality in individuals with prediabetes

There were 548 CVD death among 15,557 participants over a median follow-up of 5.5 years, the mortality rate of CVD was 6.6/1000 person-years. The corresponding mortality rates of CVD events in FPG < 5.6 mmol/L & HbA1c 5.7-6.4%, FPG 5.6-6.0 mmol/L & HbA1c < 6.5% and FPG 6.1-6.9 mmol/L & HbA1c < 6.5% groups were 7.20, 6.53 and 7.51 per 1000 person-years respectively. However, all subgroups of prediabetes were not associated with risk of CVD mortality (Table 3).

**Table 3:** Rates and hazard ratios of CVD mortality according to the blood glucose

Category	Number of events (n)	Follow up (person-years)	Rate (per 1000 person-years)	HR (95% CI)		
				Model 1	Model 2	Model 3
Normal glycemia	239	38,945.27	6.14	1	1	1
FPG < 5.6 mmol/L & HbA1c 5.7-6.4%	57	7921.65	7.20	1.11 (0.83, 1.49)	0.96 (0.72, 1.29)	1.03 (0.76, 1.38)
FPG 5.6-6.0 mmol/L & HbA1c < 6.5%	135	20,661.40	6.53	1.08 (0.87, 1.33)	1.03 (0.83, 1.27)	1.19 (0.96, 1.48)
FPG 6.1-6.9 mmol/L & HbA1c < 6.5%	117	15,583.50	7.51	1.23 (0.98, 1.53)	1.00 (0.80, 1.25)	1.15 (0.91, 1.46)

Model 1 unadjusted; Model 2 adjusted for age and sex; Model 3 adjusted for age, sex, BMI, atrial fibrillation, stroke, heart disease, triglyceride, low-density lipoprotein-cholesterol, high-density lipoprotein-cholesterol, total cholesterol, treatment for hypertension, smoking and drinking status, educational, income and physical activity

*FPG* Fasting plasma glucose, *HbA*1*c* Hemoglobin A1c

### CVD mortality in prediabetes stratified by hypertension

Given the high prevalence of hypertension (60.4%) in prediabetes individuals, we stratified the 8 subgroups according to with or without hypertension: [preDM(−)/HBP(−) as reference, preDM(−)/HBP(+), FPG < 5.6 mmol/L & HbA1c 5.7-6.4%/HBP(−), FPG 5.6-6.0 mmol/L & HbA1c < 6.5%/HBP(−), FPG 6.1-6.9 mmol/L & HbA1c < 6.5%/HBP(−), FPG < 5.6 mmol/L & HbA1c 5.7-6.4%/HBP(+), FPG 5.6-6.0 mmol/L & HbA1c < 6.5%/HBP(+), and FPG 6.1-6.9 mmol/L & HbA1c < 6.5%/HBP(+)](Table 4).

**Table 4:** Rates and hazard ratios of CVD mortality according to the blood glucose and hypertension

Outcome	Number of events (n)	Follow-up (person-years)	Rate (per 1000 person-years)	HR (95% CI)		
				Model 1	Model 2	Model 3
preDM(−)/HBP(−)	49	21,157.8	2.3	1	1	1
preDM(−)/HBP(+)	190	17,787.47	10.7	4.62 (3.37, 6.32)	2.46 (1.79, 3.38)	2.19 (1.59, 3.02)
preDM(+)/HBP(−)						
FPG < 5.6 mmol/L & HbA1c 5.7-6.4%/HBP(−)	13	3738.39	3.5	1.47 (0.80, 2.70)	1.07 (0.58, 1.98)	1.08 (0.58, 2.00)
FPG 5.6-6.0 mmol/L & HbA1c < 6.5%/HBP(−)	39	8638.27	4.5	1.98 (1.30, 3.01)	1.75 (1.15, 2.66)	1.87 (1.23, 2.85)
FPG 6.1-6.9 mmol/L & HbA1c < 6.5%/HBP(−)	14	5204.54	2.7	1.18 (0.65, 2.13)	0.89 (0.49, 1.62)	1.12 (0.62, 2.04)
preDM(+)/HBP(+)						
FPG < 5.6 mmol/L & HbA1c 5.7-6.4%/HBP(+)	44	4183.26	10.5	4.27 (2.83, 6.44)	2.30 (1.52, 3.47)	2.28 (1.50, 3.47)
FPG 5.6-6.0 mmol/L & HbA1c < 6.5%/HBP(+)	96	12,023.13	8.0	3.48 (2.47, 4.92)	2.02 (1.43, 2.85)	2.18 (1.53, 3.10)
FPG 6.1-6.9 mmol/L & HbA1c < 6.5%/HBP(+)	103	10,378.96	9.9	4.28 (3.05, 6.02)	2.25 (1.60, 3.17)	2.35 (1.65, 3.35)

Model 1 unadjusted; Model 2 adjusted for age and sex; Model 3 adjusted for age, sex, BMI, atrialfibrillation, stroke, heart disease, triglyceride, low-density lipoprotein-cholesterol, high-density lipoprotein-cholesterol, total cholesterol, treatment for hypertension, smoking and drinking status, educational, income and physical activity

*FPG* Fasting plasma glucose, *HbA*1*c* Hemoglobin A1c

**Fig. 3:**
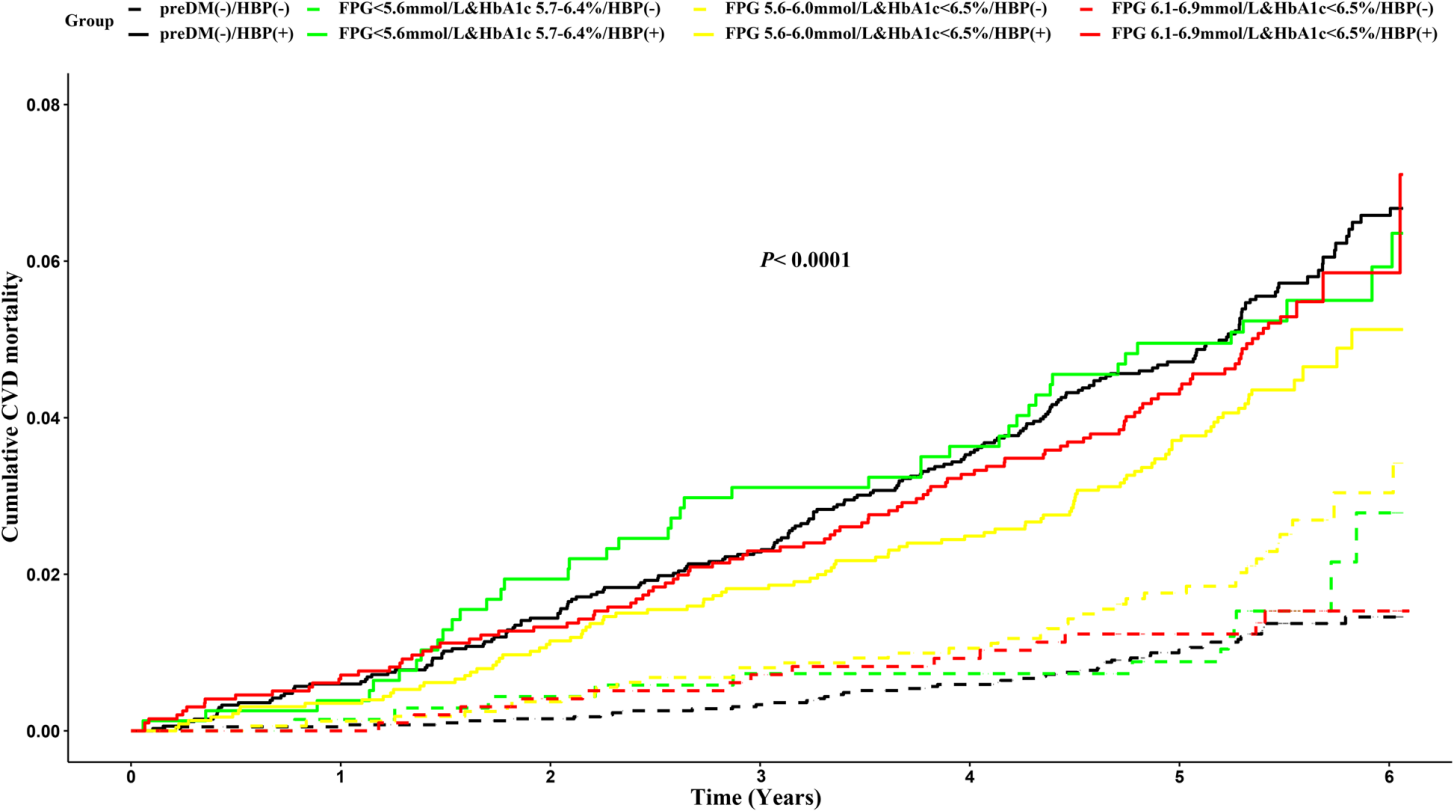
Cumulative rate of CVD mortality according to the blood glucose and hypertension at baseline

Hypertension alone group [preDM (−)/HBP (+)] increased the risk of CVD mortality, with adjusted HR of 2.19 (95% CI: 1.59, 3.02) for CVD death compared with the reference group. In contrast, isolated prediabetes groups [FPG (+) /HBP (−), HbA1c (+) /HBP (−)] were not associated with an elevated risk of CVD mortality in the fully adjusted models (all *P* > 0.05). When prediabetes is combined with hypertension, the risk of CVD death increases significantly. Multivariate-adjusted hazard ratios for CVD mortality in prediabetes subgroups combined with hypertension were as follows: 2.28 (95% CI: 1.50, 3.47) for individuals diagnosed with FPG<5.6 mmol/L & HbA1c 5.7-6.4%, 2.18 (95% CI: 1.53, 3.10) for those diagnosed with FPG 5.6-6.0 mmol/L & HbA1c<6.5%, and 2.35 (95% CI: 1.65, 3.35) for those diagnosed with FPG 6.1-6.9 & HbA1c<6.5%, compared to the reference group (Table 4). Kaplan-Meier curves also revealed that the cumulative risk of CVD mortality in the preDM(+)/HBP(+) group was substantially higher than the preDM(+)/HBP(−) group in the fully adjusted model (Fig. 3).

Moreover, when hypertension was stratified into subgroups, the CVD mortality risk increased significantly with increasing severity of hypertension (Additional Table 1). However, the rates of awareness, treatment and control of hypertension in prediabetes were frustratingly low (45.3, 35.1 and 4.8%, respectively) (Table 5).

**Table 5:** Awareness, treatment and control rates of hypertension in participants with prediabetes and subgroups

Characteristics	Prediabetes			
	FPG < 5.6 mmol/L & HbA1c 5.7-6.4%	FPG < 5.6 mmol/L & HbA1c 5.7-6.4%	FPG 5.6-6.0 mmol/L & HbA1c < 6.5%	Total
Awareness	48.3	47.1	42.7	45.3
Treatment	38.9	36.4	32.6	35.1
Control	6.9	4.4	4.4	4.8

*FPG* Fasting plasma glucose, *HbA*1*c* Hemoglobin A1c

## Discussion

The major findings of the present study are as follows: (1) The prevalence of prediabetes was 44.3% according to the ADA criteria among participants aged ≥40 years in northeastern China. (2) Prediabetes alone did not promote risk of CVD mortality. However, when prediabetes subgroups coexist with hypertension, the CVD mortality risk increased significantly. (3) Hypertension was a frequent comorbidity in prediabetes, however, the control rate of hypertension in prediabetes was dramatically low, indicating the substantial CVD risk burden in those areas.

Our results showed that the age-standardized prevalence of prediabetes was 42.8%, the prevalence was higher than the national survey (35.8%) undertaken in 2013 in the same age group and diagnostic criteria [2]. When compared to previous study reported 25.65% among adults aged ≥45 years in northwestern China [14] and other countries, such as the United States (32.74%), the prevalence of prediabetes was high in northeast China according to ADA criteria [17].

When compared to WHO criteria (FPG 6.1-6.9 mmol/L), ADA applies a lower cutoff value (FPG 5.7-6.9 mmol/L) for prediabetes. The prevalence of prediabetes increased by 20.8% using ADA fasting glucose criteria in this study, and most of population were young men living in rural areas. In 2010, ADA introduced HbA1c as a new category to diagnose prediabetes [11]. In the present study, 6.9% prediabetes were newly diagnosed after introducing HbA1c, and those subjects tended to be older women living in urban areas. Although the WHO criteria have incorporated HbA1c in the diagnosis of diabetes in 2019, the definition of prediabetes did not change, and China still adopted the WHO criteria [18]. Thus, our study suggested that HbA1c screening should be enhanced in Chinese population.

Prediabetes is a high-stake condition for diabetes, and the prevalence of diabetes in urban areas is consistently higher than in rural areas among middle-aged and elderly adults in China [19]. In contrast, our study showed that the prevalence of prediabetes was significantly higher in rural areas than in urban areas, consistent with previous studies [20]. The reason for this discrepancy is possibly due to the rapid economic progress in rural areas in recent years [21]. Coinciding with previous studies, [2, 22] we also found that men were more likely to develop prediabetes than women, and lifestyles may be responsible for these differences.

Similar to Whitehall II study, all prediabetes subgroups were not associated with an increased risk of CVD mortality in our study [12]. After stratification by hypertension, all normotensive prediabetes subgroups did not promote risk of CVD mortality compared with normotensive non-prediabetic participants. However, when prediabetes coexists with hypertension, the risk for CVD mortality increased significantly, especially when combined with grade III hypertension. Hypertension increased risk of death significantly either alone or concurrent with prediabetes, indicating hypertension plays a more significant role than prediabetes in the development of CVD mortality. The relationship between blood pressure and blood glucose remains unclear [23]. According to our study, hypertension was a frequent comorbidity in prediabetes, but the awareness, treatment and control rates among the prediabetes population were remarkably low. Thus, the lagging blood pressure management in northeast China indicated the increased cardiovascular risk in the next few decades.

Previous large-sample meta-analyses had shown that prediabetes was associated with the risk of CVD in people with or without baseline CVD, [24] and associated with increased risk and poor prognosis of heart failure, [25, 26], which further validated our findings. Compared to those studies, an advantage of our prospective study was the large-sized representative sample in northeast China, a region with a high burden of prediabetes and cardiovascular disease. Thus, our findings might provide an important guideline for the management of prediabetes patients, and might provide a reasonably reliable and relatively stable estimation of prediabetes epidemiology and CVD mortality in northeast China.

However, the present study still has several limitations. Firstly, oral glucose tolerance test (OGTT) recommended by ADA was not performed in this study, and thus impaired glucose tolerance (IGT) was not introduced to evaluate prediabetes due to lack of OGTT. The prevalence of prediabetes might be underestimated. The major reason is the difficulty of the performance of OGTT in a large sample population. Additionally, a previous study suggested that the combined application of FPG and HbA1c was associated with higher levels of detection of prediabetes compared to the joint application of FPG and 2 h PG [27]. Therefore, the combination of HbA1c and FPG was recommended in epidemiologic screening for prediabetes. Secondly, bias in the relationship between prediabetes and CVD events was inevitable due to subject loss of contact during follow-up. Thirdly, the population was recruited from one province of northeast China, the findings cannot be generalized to all participants across China.

## Conclusions

In summary, the estimated prevalence of prediabetes diagnosed by the ADA criteria was 44.3% among the participants aged ≥40 years in northeastern China. All prediabetes subgroups were not associated with an increased risk of CVD mortality, however, when hypertension was incorporated as a stratifying factor, the risk of CVD mortality elevated significantly in subjects with prediabetes and hypertension. Moreover, the CVD mortality risk was significantly increased in different FPG levels groups in prediabetes plus hypertension group. Therefore, additional HbA1c screening and lower FPG levels as well as blood pressure management strategies for prediabetes are needed in northeast China.

### Supplementary Information

**Additional file 1: Table S1.** Rates and hazard ratios of CVD mortality according to the blood glucose and severity of hypertension.

## Data Availability

The data analyzed during the current study are available from the corresponding author on reasonable request.

## Abbreviations

CVD: Cerebrovascular disease
FPG: Fasting plasma glucose
HbA1c: Hemoglobin A1c
WHO: World Health Organization
ADA: the American Diabetes Association
SBP: Systolic blood pressure
DBP: Diastolic blood pressure
AF: Atrial fibrillation
ECG: Electrocardiogram
ESH: European Society of Hypertension
CIs: Confidence intervals
HRs: hazard ratios
BMI: Body mass index
TG: Triglyceride
LDL-C: Low-density lipoprotein-cholesterol
HDL-C: High-density lipoprotein-cholesterol
TC: Total cholesterol
OGTT: Oral glucose tolerance test
IGT: Impaired glucose tolerance
